# *CACNA1C* risk variant affects facial emotion recognition in healthy individuals

**DOI:** 10.1038/srep17349

**Published:** 2015-11-27

**Authors:** Vanessa Nieratschker, Christof Brückmann, Christian Plewnia

**Affiliations:** 1 Department of Psychiatry and Psychotherapy, University of Tübingen, Calwerstrasse 14, 72076 Tübingen, Germany

## Abstract

Recognition and correct interpretation of facial emotion is essential for social interaction and communication. Previous studies have shown that impairments in this cognitive domain are common features of several psychiatric disorders. Recent association studies identified *CACNA1C* as one of the most promising genetic risk factors for psychiatric disorders and previous evidence suggests that the most replicated risk variant in *CACNA1C* (rs1006737) is affecting emotion recognition and processing. However, studies investigating the influence of rs1006737 on this intermediate phenotype in healthy subjects at the behavioral level are largely missing to date. Here, we applied the “Reading the Mind in the Eyes” test, a facial emotion recognition paradigm in a cohort of 92 healthy individuals to address this question. Whereas accuracy was not affected by genotype, *CACNA1C* rs1006737 risk-allele carries (AA/AG) showed significantly slower mean response times compared to individuals homozygous for the G-allele, indicating that healthy risk-allele carriers require more information to correctly identify a facial emotion. Our study is the first to provide evidence for an impairing behavioral effect of the *CACNA1C* risk variant rs1006737 on facial emotion recognition in healthy individuals and adds to the growing number of studies pointing towards *CACNA1C* as affecting intermediate phenotypes of psychiatric disorders.

The *CACNA1C* gene is one of the most promising candidate genes for psychiatric disorders. *CACNA1C* encodes the alpha 1C subunit of the L-type voltage-gated calcium channel and is primarily expressed in the cardiovascular system but also the brain^1^. L-type voltage-gated calcium channels play an important role in the nervous system as they assimilate the electrical activity of neurons and muscle cells in ordered to regulate physiologically relevant processes from coupling excitation to contraction in muscle cells to modulating synaptic transmission and regulating gene expression in neurons. Single nucleotide polymorphisms (SNPs) in *CACNA1C* have repeatedly been identified as risk variants for bipolar disorder, schizophrenia, and major depressive disorder in genome-wide association studies^2^,^3^,^4^,^5^,^6^,^7^. Furthermore, missense mutations in *CACNA1C* are causative for the Timothy syndrome, a multiorgan dysfunction accompanied by behavioral abnormalities including autistic traits^8^.

Impairments in emotion recognition and processing are common features of several psychiatric disorders e.g. autism spectrum disorders, bipolar disorder, schizophrenia, and major depression^9^,^10^,^11^,^12^,^13^,^14^,^15^,^16^. Several studies demonstrated an association between the most replicated *CACNA1C* risk variant rs1006737 and emotion recognition and processing, suggesting an influence of *CACNA1C* not only on the risk for psychiatric disorders themselves, but also on intermediate phenotypes underlying them. Previous findings include modified activation of several brain regions involved in emotion processing and memory formation (hippocampus, inferior occipital gyrus, fusiform gyrus, right ventrolateral prefrontal cortex, and amygdala)^17^,^18^,^19^,^20^, and decreased brain connectivity^21^. On a behavioral level, the results are more inconsistent. Two studies found delayed responses during facial emotion recognition and negative face matching in bipolar and schizophrenia patients compared to healthy control individuals^18^,^19^ but did not discover an effect of rs1006737 genotype^18^,^19^. A third study reported contradictory results. Here, an influence of *CACNA1C* rs1006737 genotype on facial emotion recognition was discovered in bipolar patients. In healthy individuals, no such effect was found^22^. Schizophrenia patients were not included in the study and the limited sample size (39 patients and 40 control individuals) might have been too small to allow the detection of effects in healthy subjects.

These previous behavioral results confirm impairments in emotion recognition and processing as an intermediate phenotype of bipolar disorder and schizophrenia, but given the limited number of studies conducted thus far, the contribution of *CACNA1C* rs1006737 to emotion recognition and processing still needs to be determined.

Therefore, we designed the present study to clarify whether an effect of *CACNA1C* on emotion recognition can also be detected at the behavioral level in healthy individuals. We hypothesized that carrier of the *CACNA1C* rs1006737 risk variant will show an impaired performance in the revised version of the “reading the mind in the eyes” task (RMET)^23^. We have chosen the RMET as it is a sensitive test which is able to reveal subtle difficulties in emotional recognition and is therefore suitable to study healthy individuals.

## Results

### Genotypes

Genotype frequency was as follows: 52 individuals where risk-allele carrier (5 AA and, 47 AG) and 40 individuals were homozygous for the G allele. Both genotype groups (AA/AG vs. GG individuals) did not differ significantly with respect to age (AA/AG: 25.808 ± 7.257, GG: 26.525 ± 5.487, p = 0.604), gender ratio (AA/AG: 51.923% male, GG: 42.5% male; p = 0.375), and education (100% secondary school education).

### Reading the mind in the eyes task (RMET)

*CACNA1C* genotype was significantly associated with performance in the RMET

**Figure 1:**
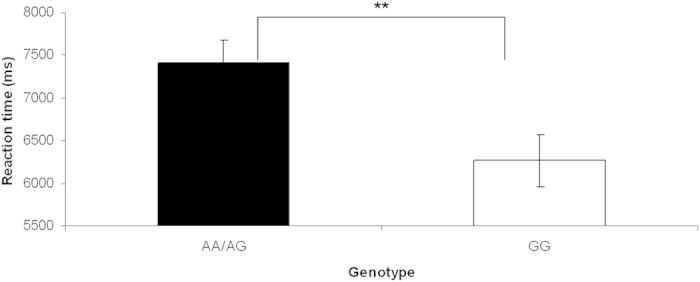
Effects of *CACNA1C* genotype on reaction time. Mean reaction time (in ms) is shown for the two genotype groups. Risk-allele carriers (AA/AG) have significantly longer mean reaction times compared to non-risk allele homozygous individuals.

**Figure 2:**
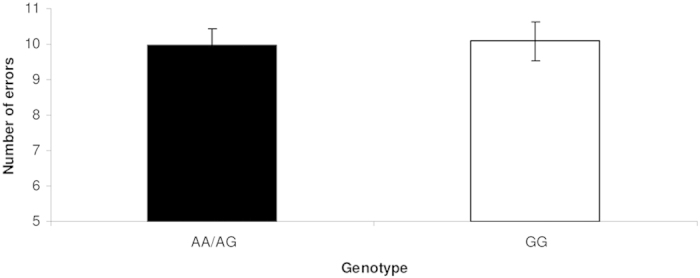
Effects of *CACNA1C* genotype on the number of errors. The number of errors is shown for the two genotype groups. *CACNA1C g*enotype had no effect on the number of errors. **p ≤ 0.01, error bars represent standard error of the mean.

[F(2,89) = 4.224, p = 0.018, partial η^2^ = 0.087]. Subsequent pairwise comparisons revealed significant longer mean reaction times in risk-allele carriers compared to non-risk allele homozygous individuals [F(1,90) = 8.129, p = 0.005, partial η^2^ = 0.083] (Fig. 1). To investigate whether the reaction time depends upon the risk-allele dose, we additionally examined the three genotype groups independently. Due to the small group size for risk-allele homozygous individuals (n = 5), the data are presented descriptively. A gene dose effect could not be observed (GG homozygous individuals [n = 40]: 6263.912 ms ± 225.884; GA heterozygous individuals [n = 47]: 7457.046 ms ± 319.317; AA homozygous individuals [n = 5]: 6965.178 ms ± 1172.396), most likely due to the small number of AA-homozygote individuals. The *CACNA1C* genotype had no effect on the number of errors [F(1,90) = 0.023, p = 0.880] (Fig. 2).

## Discussion

We investigated the influence of *CACNA1C,* a strong candidate gene for psychiatric disorders, on facial emotion recognition in healthy individuals to test its effect at the level of this intermediate phenotype. We found that carriers of the *CACNA1C* rs1006737 risk genotype (AA/AG) show a prolonged reaction time in the RMET compared to individuals homozygous for the non-risk genotype (GG), whereas the accuracy was not significantly different in both groups. Our study thus provides new evidence for an involvement of the psychiatric risk gene *CACNA1C* in cognitive processes underlying facial emotion recognition in healthy individuals.

We have chosen a cohort of healthy individuals as the investigation of genetic effects on intermediate phenotypes in non-affected individuals is a useful approach since it is not limited by medication and other confounding factors e.g. duration and severity of the illness. Facial emotion recognition can be viewed as a valid intermediate phenotype especially for bipolar disorder as previous studies showed that deficits in facial emotion recognition are present independent from mood states and are also present in healthy first degree relatives^24^, suggesting that these deficits are heritable. Our data contribute to that notion, as we were able to demonstrate, that healthy carriers of a genetic variant associated with psychiatric disorders are also impaired in their facial emotion recognition abilities.

The RMET was applied as a sensitive test suitable to investigate inter-individual differences of facial emotion recognition in healthy subjects. Deficits in this task have been observed in bipolar disorder, schizophrenia, major depression, and autism^23^,^25^,^26^,^27^. Furthermore, impaired performance in the RMET predicts higher scores on measures of autistic traits in clinical samples as well as healthy control individuals^23^,^28^.

In this study, we only investigated the effects of the most replicated and best described variant in *CACNA1C* (rs1006737) without considering the contribution of other polymorphisms in *CACNA1C* or additional genes potentially interacting with *CACNA1C*. Rs1006737 is located in an intron and is therefore not causing an amino acid substitution in the encoded protein. Nevertheless, rs1006737 potentially has a functional impact, as a proxy variant for rs1006737 (rs2159100) is associated with increased gene expression in the dorsolateral prefrontal cortex^17^. We cannot establish definitively whether the effect we observed in our study can be attributed to rs1006737, the proxy variant rs2159100, or any other genetic variant in linkage disequilibrium to rs1006737.

A further limitation of our study is that we cannot distinguish whether the observed prolonged reaction time in the RMET is based on an impairment specific for emotional recognition or rather reflects a more general underlying deficit in complex signal detection and processing as we did not check for slower reaction times of risk-allele carriers in other neuropsychological tasks. Healthy carriers of the risk genotype were already found to be impaired in alerting and orienting^29^, in verbal fluency^30^, and in working memory^31^. However, other studies contradict these findings^22^,^32^ or did not find impairments of healthy *CACNA1C* risk genotype carriers in other aspects of cognition for instance in attention^32^, verbal learning and memory^32^,^33^,^34^,^35^,^36^ or logical memory^37^.

We have decided to take the mean reaction time for all trials including errors, to give a valid estimate of the processing time a subject requires to identify and react to a facial emotion. Delays in this cognitive domain can severely impair social interactions and competence and are associated with core symptomatology especially in schizophrenia^38^. For instance, it has been shown that schizophrenia patients require more visual information, and therefore more time to correctly identify emotional expression in faces, compared to controls^39^,^40^. Therefore, differences in emotion recognition between the risk-allele carrier and the other subjects may be more prominent in speed of performance than in accuracy of the RMET^41^. Since we did not detect differences in accuracy, our data most likely also reflect differences in the amount of information needed to correctly identify a facial emotion. In healthy individuals, this characteristic is not associated with obvious impairments. However, in complex and challenging situations, this subtle impairment might significantly exert negative effects on social interactions, and in psychiatric patients it might add to further deficits and contribute to psychosocial dysfunction.

The effect of *CANA1C* on facial emotion recognition is most likely not specific, as deficits in a variety of other intermediate phenotypes have been reported in *CANA1C* risk-allele carriers (reviewed in^42^,^43^). However, our data provide clear support that *CACNA1C* has a subtle but functionally relevant influence on facial emotion recognition as an intermediate phenotype involved in psychiatric disorders.

In conclusion, the present study is the first to provide evidence for an impairing effect of the *CACNA1C* risk genotype rs1006737 AA/AG on facial emotion recognition in healthy individuals. Our finding that risk-allele carriers are subtly challenged in facial emotion recognition adds to the growing body of knowledge on the role of *CACNA1C* in cognitive functions affected in various psychiatric disorders. However, future studies are needed to replicate our finding in independent cohorts as well as to investigate the specificity of the effect.

## Material and Methods

### Subjects

Ninety-two healthy volunteers (44 males, 48 females; mean age = 26.12, SD = 6.523) participated in this study and gave written informed consent to the experimental procedure approved by the University of Tübingen local ethics committee. All experiments were carried out in accordance with the approved guidelines. None of the subjects had a history of physical, mental, or neurological illness and none of the subjects had performed the “Reading the mind in the eyes” (REM) task before.

### Reading the mind in the eyes task (RMET)

The RMET was used to assess emotional recognition. The task was conducted as previously described^23^. In brief, 36 black-and-white photographs (15 cm × 6 cm) of the area of the face including and immediately surrounding the eyes were presented together with a word correctly characterizing the emotional state of the person in the photograph as well as three distracter words of the same emotional valence. Participants were asked to choose the most suitable adjective and to quickly click it on the screen. The next picture appears immediately after the response. The number of incorrect choices was measured as number of errors, the time interval between presentation of the photograph and the click on the word was recorded as reaction time.

### Genotyping

Genomic DNA was extracted from ethylenediaminetetraacetic acid (EDTA) anti-coagulated venous blood according to standard protocols. *CACNA1C* rs1006737 was genotyped on a StepOne system (life technologies; Darmstadt; Germany) using TaqMan®SNP Genotyping Assay C___2584015_10 (life technologies; Darmstadt; Germany) and the standard protocol for allelic discrimination. Accuracy was assessed by duplicating 15% of the original sample, and reproducibility was 100%. The genotype frequencies did not deviate from Hardy–Weinberg equilibrium (HWE; p = 0.06).

### Statistical analysis

Number of errors and mean reaction time were treated as outcome variables. The data were analysed with SPSS (IBM SPSS Statistics 22.0; Ehningen; Germany). The effect of rs1006737 on the outcome variables was evaluated by applying a multivariate analysis of variance (MANOVA) with the between factor *genotype*_*A allele carrier vs. G homozygout*_. Results were considered significant when p ≤ 0.05. For significant interaction F-values, *post hoc* pairwise comparisons were performed.

## Additional Information

**How to cite this article**: Nieratschker, V. *et al. CACNA1C* risk variant affects facial emotion recognition in healthy individuals. *Sci. Rep.* **5**, 17349; doi: 10.1038/srep17349 (2015).
